# Need for additional capacity and improved capability for molecular detection of yellow fever virus in European Expert Laboratories: External Quality Assessment, March 2018

**DOI:** 10.2807/1560-7917.ES.2018.23.28.1800341

**Published:** 2018-07-12

**Authors:** Cristina Domingo, Heinz Ellerbrok, Marion Koopmans, Andreas Nitsche, Katrin Leitmeyer, Rémi N. Charrel, Chantal B.E.M. Reusken

**Affiliations:** 1 Highly Pathogenic Viruses (ZBS 1), Centre for Biological Threats and Special Pathogens, Robert Koch Institute, World Health Organization (WHO) Collaborating Centre for Emerging Infections and Biological Threats, Berlin, Germany; 2 Department of Viroscience, World Health Organization (WHO) Collaborating Centre for Arbovirus and Haemorrhagic Fever Reference and Research, Erasmus MC, Rotterdam, The Netherlands; 3 European Centre for Disease Prevention and Control (ECDC), Solna, Sweden; 4 Institute of Research and Development, Unit of Emerging Viruses (UMR), Faculty of Medicine, Aix Marseille University, Marseille, France

**Keywords:** yellow fever virus, laboratory surveillance, emerging or re-emerging diseases, imported viral diseases, vector-borne infections

## Abstract

An external quality assessment of yellow fever virus (YFV) molecular detection in European laboratories was organised in rapid response to an increase in human cases in Brazil in 2018 with risk of import to Europe. Detection of YFV was assessed among 32 laboratories in 23/31 European Union (EU) and European Economic Area (EEA) countries and two laboratories in one non-EU/EEA country. Adequate capabilities were lacking in 10/23 countries; five did not participate as they lacked implemented assays.

In March 2018, the Emerging Viral Diseases-Expert Laboratory Network (EVD-LabNet), funded by the European Centre for Disease Prevention and Control (ECDC) in Stockholm, Sweden, organised an external quality assessment (EQA) of molecular detection of yellow fever virus (YFV). The EQA was a rapid response to the recent outbreaks with YFV in South America and the increasing number of unvaccinated citizens from European Union /European Economic Area (EU/EEA) countries that acquired infection while travelling to outbreak regions [1-3].

## Participation of European Expert Laboratories

In total, 71 laboratories were invited to participate in the EQA: 60 laboratories in 30 EU/EEA countries (excluding Liechtenstein, which has no EVD-LabNet member laboratory), seven laboratories in seven EU pre-accession countries and four laboratories in two other European countries. Thirty-two laboratories in 23 EU/EEA countries and two laboratories in one other European country participated in the EQA (Figure). Of the seven EU/EEA countries that did not participate, five laboratories indicated that they did not have a test available at the time of the EQA, one laboratory had no funds and one laboratory had no permission to participate. Of the seven invited laboratories in the EU pre-accession countries, five had no test available and two did not indicate a reason for not participating.

**Figure:**
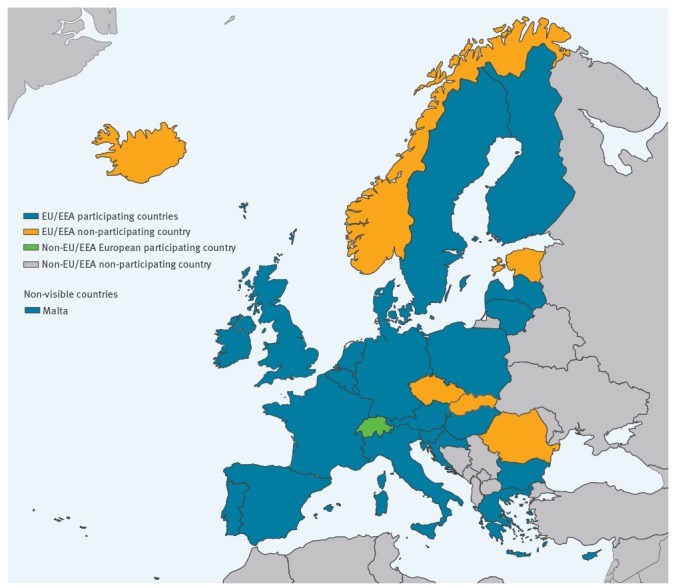
Overview of the number of Emerging Viral Diseases-Expert Laboratory Network laboratories per country that participated in the yellow fever virus molecular external quality assessment, March 2018 (n = 34)

EU/EEA participating countries: Italy 6 laboratories; Germany 4 laboratories; Spain 2 laboratories; all other countries 1 laboratory. Non-EU/EEA participating countries: Switzerland 2 laboratories.

## External quality assessment set-up

For the preparation of the EQA panel, Vero E6 cell culture supernatants were infected with different YFV strains (vaccine strain YFV-17D, South American strain genotype 1E Brazil 2008, West African strain Ivory Coast 1999, GenBank AY603338), inactivated by heat (56 °C, 1 h) and gamma irradiation (25 kilogray) and tested for non-infectivity. Plasma or urine was spiked to prepare a set of 12 representative samples (Table 1). As a specificity control, plasma samples containing inactivated lineage 1 West Nile virus (WNV, strain New York), the four dengue virus serotypes (DENV-1, strain Thai 1958; DENV-2, TH-36 strain; DENV-3, H87 strain; DENV-4, H241 strain), and Zika virus (ZIKV, strain MR766) and a negative control (plasma and urine) were included (Table 1). The samples were number coded, freeze-dried (Christ, AlphaI-5, Hanau, Germany) and stored at 4 °C until dispatch. Sample quality and YFV load were estimated in two different panels using a reference real-time quantitative polymerase chain reaction (RT-qPCR) [4], and by a pan-flavirus RT-qPCR [5]. The non-YFV viral loads were determined using RT-qPCR (WNV) [6] and in house RT-qPCRs (DENV and ZIKV). The panel was further validated by two independent reference laboratories using a YFV specific RT-qPCR [4,7]. On 26 February, the freeze-dried panels were shipped at room temperature to the participants and all reported the panel to be in good condition on arrival. EQA results could be submitted until 31 March 2018 via an online system.

**Table 1:** Composition and panel results of the yellow fever virus molecular detection external quality assessment, March 2018 (n = 46)

Parameter	Sample number	Virus (*Strain*)	Viral RNA copies/sample	Matrix	Correct result	Inconclusive result	False negative result	False positive result	Flavivirus not-specified	Total % of test with correct result
Sensitivity	8	YFV American (Brazil)	2.8E + 06	Plasma	35	1	7	NA	3	76
	9	YFV American (Brazil)	2.6E + 05	Plasma	35	1	8	NA	3	76
	2	YFV American (Brazil)	3.5E + 04	Plasma	33	1	9	NA	3	71.7
	3	YFV American (Brazil)	3.4E + 03	Plasma	28	2	13	NA	3	60.8
	5	YFV American (Brazil)	3.5E + 05	Urine	34	1	8	NA	3	73.9
	11	YFV American (Brazil)	2.3E + 03	Urine	25	1	17	NA	3	54.3
	6	YFV Africa (Ivory Coast 1999)	4.7E + 06	Plasma	35	1	7	NA	3	76
	12	YFV Africa (Ivory Coast 1999)	4.7E + 05	Plasma	34	1	8	NA	3	73.9
	15	YFV Africa (Ivory Coast 1999)	4.9E + 04	Plasma	32	1	10	NA	3	69.5
	13	YFV Africa (Ivory Coast 1999)	3.7E + 03	Plasma	33	1	9	NA	3	71.7
	14	YFV-vaccine (*17D*)	6.5E + 04	Plasma	38	0	5	NA	3	82.6
	1	YFV-vaccine (*17D*)	5.4E + 03	Plasma	32	2	10	NA	2	69.5
Specificity		ZIKV (*MR766*) DENV-1 (*Thailand 1958*)	4.2E + 05 3.5E + 06							
		DENV-2 (*TH-36*)	2.2E + 06							
	4	DENV-3 (*H-87*)	3.9E + 05	Plasma	40	2	NA	2	3	86.9
		DENV-4 (*H241*)	6.6E + 04							
		WNV (*New York*)	1.27 + 04							
Contamination control	7	Negative	NA	Plasma	43	0	NA	2	1	93.4
	10	Negative	NA	Urine	46	0	NA	0	0	100

DENV: dengue virus; NA: not applicable; WNV: West Nile virus; YFV: yellow fever virus; ZIKV: Zika virus.

## Outcomes

Thirty-four laboratories submitted results for a total of 46 panel tests. There were 20 different in-house (including own design) and four commercial RT-PCRs represented in the EQA; 15 YFV-specific, three vaccine-strain specific and six pan-flavivirus RT-PCRs. In 10 of 13 laboratories submitting results based on a pan-flavivirus RT-PCR, this RT-PCR was combined with amplicon sequencing to determine the specific flavivirus involved (Table 2).

**Table 2:** Overview of RT-PCR systems for yellow fever virus detection used by 34 laboratories in the Emerging Viral Diseases-Expert Laboratory Network external quality assessment of molecular detection of yellow fever virus, March 2018 (n = 46)

RT-PCR used and specificity	YFV genome target	number of laboratories
**In-house YFV specific (wildtype/ vaccine strain)**		
Domingo et al. 2012 [4]	5'-UTR	4
Drosten et al. 2002 [7]	5'-UTR	7
Fischer et al. 2017 [13]	NS1	2
Weidmann et al. 2010 [14]	5'-UTR	3
Own design	not specified	7
Own design-adapted from [4]	5'-UTR	1
Fast Track diagnostics Tropic fever Africa (commercial)	unknown	1
Genesig (commercial)	unknown	1
Real Star YFV RT-PCR kit 1.0 (commercial)	unknown	1
**In-house YFV vaccine strain specific**		
Bae et al., 2003 [8]	NS3	2
Fernandes-Monteiro et al. 2015 [10]	NS5	1
Mantel et al. 2008 [9]	NS5	2
**Pan-flavivirus**		
Ayers et al. 2006 + seq [15]	NS5	1
Moureau et al. 2007 + seq [16]	NS5	1
Patel et al. 2013 + seq [17]	NS5	3
Scaramozzino et al. 2001 + seq [18]	NS5	4
Own design (pan-flavivirus) + seq	unknown	1
Patel et al. 2013 [17]	NS5	1
Scaramozzino et al. 2001 [18]	NS5	1
Genekam (commercial pan-flavivirus)	unknown	1
**Other, no YFV detection**		
Fast Track diagnostics Tropic fever Asia	NA	1
**Total number of submitted tests**	**NA**	**46**

NA: not applicable; YFV: yellow fever virus.

Twenty-three laboratories used one method for detection, while 10 used a combination of two methods and one laboratory combined three methods (Supplement). For the laboratories that used more than one test, the submitted results could have represented a routine combinatorial diagnostic approach e.g. combining a YF-17D specific with a YFV wildtype-specific PCR assay. Although the individually reported assays could not give a perfect overall result, the logical combination of more than one assay might give a satisfactory end result at the laboratory level.

At the laboratory level 18 of 34 participating laboratories had at least one test that scored the presence/absence of YFV RNA in the panel 100% correctly (Category I Supplement). At the country level this corresponded to 13 of 23 EU/EEA countries and one non-EU/EEA European country. A within-laboratory combination of test results did not yield any additional laboratories with a result that was 100% correct. These results indicate that there is a definite need for improvement of current YFV molecular diagnostics in 16 of the 34 participating laboratories and in 10 of the 23 participating EU/EEA countries respectively. At the test level, 20 of 46 submitted test results were 100% correct (Supplement).

Samples containing YF wildtype strains were missed by the three vaccine-strain-specific RT-PCR tests (Table 2, Supplement) [8-10]. Of the five laboratories submitting results based on YFV 17D-strain-specific tests, two laboratories in one EU/EEA country did not have an alternative or complementary parallel test available; meaning that these laboratories would miss YF in travellers returning from regions with YFV activity.

As all the YFV positive samples in the panel were representative for the range of viremia observed in clinical samples of acute YFV cases [11,12] it highlights the need for improvement of sensitivity in all laboratories that missed the samples with lower RNA loads (Category II Supplement). Furthermore, combinational testing by laboratories showed different performances related to the sensitivity of the individual assays and/or the performance across the laboratories.

Differences in the detection of the samples as an effect of virus strain were not significant and were mostly related to the characteristics of the assay used (Table 2, Supplement).

Among the exceptions, was one laboratory that missed all wild-type YFV samples using RT-PCR systems based on Drosten et al. [8] and Patel et al. [12]. This could imply the issues were at a laboratory-specific level, as multiple laboratories analysed the complete panel correctly based on these RT-PCR tests (Supplement). Another laboratory missed all YFV RNA positive samples (both vaccine strain and wildtype YFV based) as they used a commercial kit that does not detect YFV. One laboratory had only capacity for pan-flavivirus testing without sequencing and could not distinguish between the YFV RNA positive samples and the sample containing a mixture of flavivirus RNA. Finally, four laboratories indicated to have detected YFV RNA in one or more YFV RNA negative samples which is suggestive for contamination issues.

## Discussion

Since December 2017, Brazil has experienced an increase in YFV cases with 1,266 confirmed and 1,232 suspected human cases in the period 1 July 2017 to 16 May 2018 [19]. The majority of notifications were from January to April 2018. The appearance of epizootics in non-human primates and of the first human cases in close vicinity to the metropolitan areas of Sao Paulo and Rio de Janeiro in early 2018, raised concerns about higher risks of exposure for international travellers [1,3]. On 15 January 2018, the first imported case related to the Sao Paulo metropolitan area was reported from the Netherlands [2] in a returning traveller showing the potential risk of international spread [20]. It was estimated that 1.2 million travellers would return from Brazil to Europe during the peak of the YFV season (December to May) [3], therefore, on 25 January 2018, the ECDC asked EVD-LabNet to assess the quality of YFV molecular diagnostics in its member laboratories. During and after the EQA, there have been eight additional YFV cases imported to Europe; one to the Czech Republic, two to France, three to Germany (one of which reported by UK), one to Romania and one to Switzerland [21]. This warrants the need for a reliable diagnostic capability and adequate capacity to support individual patient care, surveillance and response activities in Europe.

Of the 30 EU/EEA countries, five non-participating countries indicated that they have no test, two non-participating countries had a test available and 10 of the 23 participating countries showed insufficient capability for molecular testing for YFV. This indicates room for improvement in at least half of the countries. As this EQA assessed the diagnostic workflow as a whole from sample receipt to result reporting, it is not possible to recommend specific RT-PCR tests over others, as the influence of e.g. nucleic acid extraction methodology cannot be excluded.

Two laboratories only applied a vaccine strain-specific RT-PCR, meaning that they would miss YF cases in returning travellers. This issue was already noted in a previous YF EQA [4] and could be solved by the laboratories through use of another primary assay or using a complementary one. These laboratories were located in a country with endemic presence of *Ae. albopictus* that is a potential vector for YFV transmission [22-24]. One laboratory only had capacity for pan-flavivirus testing without sequencing, which without specific follow-up could potentially create interpretation issues in returning travellers presenting with other flavivius diseases or in areas where flaviviruses are endemic; DENV and YFV have an overlapping clinical manifestation and geographic distribution [25]. WNV, tick-borne encephalitis virus (TBEV) and Usutu virus are endemic in partially overlapping areas in Europe [26]. This laboratory would benefit from pre-arranged access to confirmatory testing.

The variety in RT-PCR systems used in the EQA reflects the absence of standardisation in wildtype YFV diagnostics across European laboratories potentially hampering consistent case finding and reporting.

The results of the EQA were reported back to the individual laboratories in April 2018. Within one month, three laboratories in three EU/EEA countries informed the EVD-LabNet that they have taken successful actions to improve their capability. New protocols have been shared with two laboratories underlining the value of EQA exercises in laboratory preparedness and response activities.
