# Authors’ correction for Euro Surveill. 2018;23(28)

**DOI:** 10.2807/1560-7917.ES.2018.23.40.1810042

**Published:** 2018-10-04

**Authors:** Eurosurveillance editorial team

**Affiliations:** 1 European Centre for Disease Prevention and Control (ECDC), Stockholm, Sweden

As requested by the authors of the article ‘Need for additional capacity and improved capability for molecular detection of yellow fever virus in European Expert Laboratories: External Quality Assessment, March 2018’ published on 12 July 2018, the colour blue for Malta (EU/EEA participating country), was changed to orange (EU/EEA non-participating country) in the figure legend. The mistake was corrected on 2 October 2018.

